# Characterization of pyoverdine and achromobactin in *Pseudomonas syringae *pv. phaseolicola 1448a

**DOI:** 10.1186/1471-2180-11-218

**Published:** 2011-10-03

**Authors:** Jeremy G Owen, David F Ackerley

**Affiliations:** 1 School of Biological Sciences, Victoria University of Wellington, Kelburn Parade, PO Box 600, Wellington 6140, New Zealand; 2 Howard Hughes Medical Institute, Laboratory of Genetically Encoded Small Molecules, The Rockefeller University, 1230 York Avenue, New York, NY 10065, USA

## Abstract

**Background:**

*Pseudomonas syringae *pv. phaseolicola 1448a (*P. syringae *1448a), the causative agent of bean halo blight, is a bacterium capable of occupying diverse biological niches. Under conditions of iron starvation *P. syringae *1448a secretes siderophores for active uptake of iron. The primary siderophore of *P. syringae *1448a is pyoverdine, a fluorescent molecule that is assembled from amino acid precursors by non-ribosomal peptide synthetase (NRPS) enzymes. Whereas other species of *Pseudomonas *often exhibit structural variations in the pyoverdine produced by different strains, all *P. syringae *pathovars previously tested have been found to make an identical pyoverdine molecule. *P. syringae *1448a also appears to have the genetic potential to make two secondary siderophores, achromobactin and yersiniabactin, each of which has previously been detected in different *P. syringae *pathovars.

**Results:**

Five putative pyoverdine NRPS genes in *P. syringae *1448a were characterized *in-silico *and their role in pyoverdine biosynthesis was confirmed by gene knockout. Pyoverdine was purified from *P. syringae *1448a and analyzed by MALDI-TOF and MS/MS spectroscopy. Peaks were detected corresponding to the expected sizes for the pyoverdine structure previously found in other *P. syringae *pathovars, but surprisingly *P. syringae *1448a appears to also produce a variant pyoverdine species that has an additional 71 Da monomer incorporated into the peptide side chain. Creation of pyoverdine null mutants of *P. syringae *1448a revealed that this strain also produces achromobactin as a temperature-regulated secondary siderophore, but does not appear to make yersiniabactin. Pyoverdine and achromobactin null mutants were characterized in regard to siderophore production, iron uptake, virulence and growth in iron limited conditions.

**Conclusions:**

This study provides the first evidence of a *P. syringae *pathovar producing a side chain variant form of pyoverdine. We also describe novel IC_50 _and liquid CAS assays to quantify the contribution of different siderophores across a range of iron starvation conditions, and show that although achromobactin has potential to contribute to fitness its contribution is masked by the presence of pyoverdine, which is a significantly more effective siderophore. Neither pyoverdine nor achromobactin appear to be required for *P. syringae *1448a to cause bean halo blight, indicating that these siderophores are not promising targets for crop protection strategies.

## Background

Acquisition of iron is essential for growth of most bacteria. However, due to insolubility at neutral pH the bioavailability of iron is extremely low in most natural environments. To circumvent this problem many bacteria respond to iron starvation by synthesizing high affinity iron-chelating molecules known as siderophores. These siderophores are secreted into the extra-cellular environment where they bind ferric iron and are then actively transported back into the cell via specific ferric-siderophore receptors [1]. Siderophores play a prominent role in the biology of fluorescent pseudomonads, a genus renowned for occupying a very wide range of environmental niches. Fluorescent pseudomonads synthesize the peptide-derived molecule pyoverdine as their primary siderophore, together with secondary siderophores that have lower affinity for iron [2]. Although pseudomonads are not obligate pathogens, many species are capable of causing disease in a wide variety of hosts [3,4]. As iron restriction is a key host defense mechanism, pyoverdine is frequently implicated as an important virulence factor [5,6].

Pyoverdine is synthesized from amino acid precursors by non-ribosomal peptide synthetase enzymes (NRPS) [7,8]. It is pyoverdine that provides the fluorescent *Pseudomonas *species with their defining fluorescence and yellow-green pigmentation under conditions of iron limitation [9]. These properties derive from an invariant dihydroxyquinoline chromophore, to which is attached an acyl moiety and a strain-specific peptide side chain [10]. More than 50 different pyoverdine structures have been described to date [11] and the variability of the peptide side chain of pyoverdines from different strains reflects rapid evolution of both the NRPS that synthesize this side chain and the outer membrane receptors that recognize ferric pyoverdine [12]. Analysis of the pyoverdine locus of different *P. aeruginosa *strains indicated that it is the most divergent region in the core genome and that its evolution has been substantially shaped by horizontal gene transfer [12,13]. The diversification of pyoverdine structures is particularly interesting when viewed in the context of NRPS manipulation experiments [14-16] - the wide variety of pyoverdine structures that has resulted from natural recombination of a limited pool of NRPS modules provides clues as to how nature has overcome the barriers that frequently limit artificial recombination of NRPS enzymes [16,17]. Moreover, the ability to detect pyoverdine production at nanomolar levels by UV-fluorescent screening [18] makes the pyoverdine synthetases potentially a very attractive model system to study NRPS recombination. However, in terms of providing 'raw material' for such work, the only biochemical analysis of a pyoverdine NRPS to date focused on the L-threonine incorporating enzyme PvdD of *P. aeruginosa *PAO1 [19]. In the work described here we aimed to expand this focus to the NRPS enzymes of another fluorescent pseudomonad, *Pseudomonas syringae *pv. phaseolicola 1448a (*P. syringae *1448a), which secretes an alternative form of pyoverdine to PAO1.

During the course of this study, pyoverdine null mutants were generated, revealing that *P. syringae *1448a (like *P. syringae *pathovars syringae B728a [20], syringae 22d/93 [21], and glycinea 1a/96 [21]) produces achromobactin as a secondary siderophore. In contrast to pyoverdine, achromobactin is synthesized by a mechanism that is entirely independent of NRPS enzymes [22]. NRPS-independent siderophores have been studied far less intensively than their NRPS-dependent counterparts, and their mechanisms of synthesis have only recently begun to be deciphered. Three types (A, B and C) of NRPS-independent siderophore synthetase enzymes have been identified to date, each responsible for the attachment of a different functional group to a citric acid backbone [22,23]. The achromobactin biosynthetic pathway is a particularly valuable resource for the study of these enzymes as it relies on the action of all three types of synthetase [22,24]. Achromobactin has been shown to be important for virulence in *Dickeya dadantii *(formerly *Erwinia chrysanthemi*) [25], and both pyoverdine and achromobactin contribute to epiphytic fitness of *P. syringae *pv. syringae 22d/93 [21], but the contribution of siderophores to virulence of *P. syringae *1448a has not previously been characterized. We therefore examined the roles of both achromobactin and pyoverdine in virulence of *P. syringae *1448a, as well as their relative contribution to iron uptake and growth under more precisely defined conditions.

## Results

### Identification and *in silico *characterization of the *P. syringae *1448a pyoverdine locus

The biosynthesis of pyoverdine has been most extensively studied in *P. aeruginosa *PAO1 and most, if not all, of the genes required for pyoverdine synthesis in this strain have now been identified [6,10,26]. Ravel and Cornelis [8] used the PAO1 pyoverdine genetic locus as a blueprint for annotation of the pyoverdine loci from three other fluorescent pseudomonads, including *P. syringae *pv. tomato DC3000. We adopted a similar strategy to interrogate the *P. syringae *1448a genome, individually BLASTP searching all of the known PAO1 pyoverdine proteins against the *P. syringae *1448a sequence database [27].

**Figure 1:**
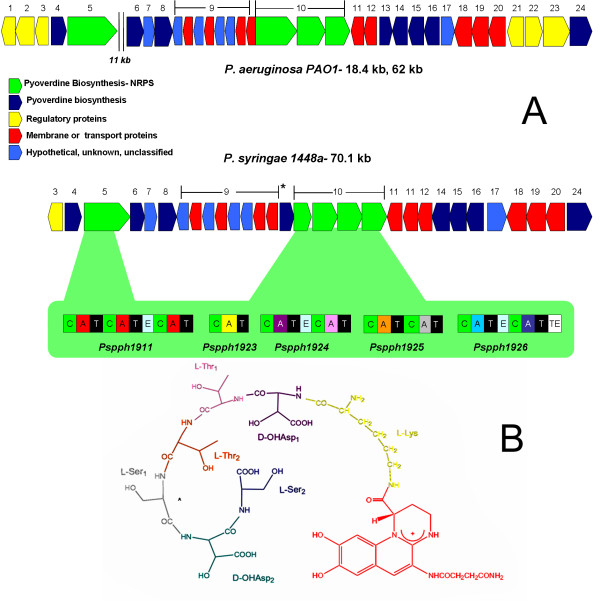
**Comparison of the pyoverdine loci of *P. aeruginosa* PAO1 and *P. syringae* 1448a**. A. The core PAO1 pyoverdine genes fall into two closely linked clusters, 11 kb apart. In contrast, the core *P. syringae* 1448a genes form a single contiguous cluster. Genes are color coded according to their function, as indicated in the key; and orthologous genes in each organism have been assigned the same number, corresponding to the annotations in Table 1. The green highlighted region details the modular structure of the *P. syringae* 1448a pyoverdine NRPS genes (C = condensation domain, A = Adenylation domain, T = Thiolation domain, E = Epimerization domain, TE = Thioesterase domain). A-domains are color coded to correspond with the amino acid residue that each incorporates into the *P. syringae* 1448a pyoverdine molecule (as pictured in **B**).

The genomic organization of pyoverdine genes in *P. syringae *1448a is highly similar to the *P. syringae *DC3000 genetic locus presented by Ravel and Cornelis [8], but less similar to that of PAO1 (Figure 1A, Table 1). Given the similarity with the *P. syringae *DC3000 genetic locus and the excellent earlier analysis of Ravel and Cornelis, we confine our analysis of the non-NRPS genes of *P. syringae *1448a to two aspects not previously noted by them. The first concerns the only PAO1 gene that clearly lacks an ortholog in *P. syringae, pvdF*, which encodes an enzyme required for generating the N^5^-formyl-N^5^-hydroxyornithine residues that are present in the PAO1 (but not *P. syringae*) pyoverdine side chain. Instead, *P. syringae *1448a contains a gene (*Pspph*1922; marked * in Figure 1A) that is 37% identical at a predicted protein level to the *syrP *gene of *Pseudomonas syringae *pv. syringae. Originally mis-annotated as a putative regulatory gene, SyrP has subsequently been shown to be an aspartate hydroxylase that is required for synthesis of the NRPS-derived phytotoxin syringomycin [28]. On this basis we propose that *Pspph*1922 very likely catalyzes β-hydroxylation of two hydroxyaspartate residues expected to be present in the *P. syringae *1448a pyoverdine side chain (Figure 1B), with equivalent iron-chelating roles to the N^5^-formyl-N^5^-hydroxyornithine residues of PAO1 pyoverdine. We also note that *P. syringae *1448a contains two orthologs of the PAO1 ferripyoverdine receptor gene *fpvA*. The predicted products of these genes share 52.5% amino acid identity with one another, and 35.5% (*Pspph*1927) and 36.0% (*Pspph*1928) with FpvA from PAO1. PAO1 itself contains a second type I ferripyoverdine receptor gene, *fpvB*, whose product is 54% identical to FpvA [29]; however in PAO1 this second ferripyoverdine receptor gene lies outside the pyoverdine locus.

**Table 1:** Summary of PAO1 and Ps1448a pyoverdine gene alignment results

	PAO1 gene	Function in *P. aeruginosa *PAO1	**Ps1448a ortholog(s)**^†^
**1**	*pvdY*	Regulatory protein	1515
**2**	*pvdX*	Regulatory protein	3568
**3**	*pvdS*	ECF iron sigma factor	**1909**
**4**	*pvdG*	Thioesterase (34% identity with GrsT thioesterase from *Bacillus brevis*)	**1910**
**5**	*pvdL*	Chromophore peptide synthetase	**1911**
**6**	*pvdH*	Aminotransferase	**1912**
**7**	*Pa2412*	MbtH-like protein (no known function)	**1913**
**8**	*Pa2411*	Thioesterase (36% identity with thioesterase GrsT from *Bacillus brevis*)	**1910**
**9**	*Pa2403-2410*	No known function, however expression of these genes is co-regulated with pyoverdine synthesis genes. 2408 and 2409 are predicted to encode an ABC transporter	**1914-1921**
*	Not present	(Likely pyoverdine aspartate hydroxylase of Ps1448a)	**1922**
**10**	*pvdDIJ*	Pyoverdine side chain NRPS	**1923-1926**
**11**	*fpvA*	Ferripyoverdine receptor protein	1870, **1927, 1928**
**12**	*pvdE*	ABC transporter (secretion)	**1929**
**13**	*pvdF*	N5-hydroxyornithine transformylase	Not present
**14**	*pvdO*	No known function	**1930**
**15**	*pvdN*	26% identity with isopenicillin N epimerase from *Streptomyces clavuligerus*	**1931**
**16**	*pvdM*	Dipeptidase (23% identity with porcine dipeptidase)	**1932**
**17**	*pvdP*	No known function	**1933**
**18**	*Pa2391*	Porin (over 30% identity with outer membrane factor (OMF) proteins of RND/MFP/OMF-type efflux systems)	**1934**
**19**	*Pa2390*	ABC transporter (over 40% identity with resistance-nodulation-division (RND)-type transporter components of RND/MFP/OMF-type efflux systems)	**1935/*macB***
**20**	*Pa2389*	Periplasmic protein (over 30% identity with periplasmic membrane fusion proteins (MFP) of RND/MFP/OMF-type efflux systems)	**1936**
**21**	*fpvR*	Antisigma factor for PvdS and FpvI	2117, 4764
**22**	*fpvI*	ECF sigma factor required for expression of *fpvA*	4765, 1175, 1093, 2747, **1909**
**23**	*pvdA*	L-ornithine hydroxylase	2415, 3753
**24**	*pvdQ*	Acylase (38% identity with Aculeacin A acylase from *Actinoplanes utahensis*)	**1937**

†Gene numbers for Ps1448a are as annotated in the Pseudomonas genome database. Genes were presumed to be orthologs if they belonged to the same COG group. Hits are listed in order of significance, with those falling within the Ps1448a pyoverdine locus (as pictured in figure 1) listed in bold.

*P. syringae *1448a also contains 5 NRPS genes that lie within the pyoverdine locus (Figure 1A). The gene *Pspph*1911 presumably governs synthesis of the pyoverdine chromophore, as it shares 72.4% predicted amino acid identity with the chromophore NRPS gene *pvdL *of *P. aeruginosa *PAO1 and homologs of this gene are present in all fluorescent pseudomonads that have been examined [10,30,31]. Likewise, the four contiguous genes *Pspph*1923-1926 are expected to encode the side chain NRPS of *P. syringae *1448a, and the total number of NRPS modules in these genes (7) corresponds exactly with the number of amino acids in the *P. syringae *1448a pyoverdine side chain. Bioinformatic prediction of the substrate specificity of these modules (using the online NRPS analysis tool http://nrps.igs.umaryland.edu/nrps/[32]) as well as heuristic prediction software [33] revealed that their likely substrates are (in linear order) L-Lys, D-Asp, L-Thr, L-Thr, L-Ser, D-Asp, L-Ser (Table 2) (stereospecificity being assigned on the basis of E-domain presence or absence in that module). Assuming β-hydroxylation of the two D-Asp residues as noted above, and the co-linearity that is typical of NRPS clusters [34], this substrate specificity is consistent with the linear order of residues identified in the pyoverdine side chains of several other *P. syringae *pathovars [35,36] (Figure 1B).

**Table 2:** In silico prediction of A-domain specificity for Ps1448a pyoverdine side chain NRPS

A domain	8 residue signature alignment	Identity of best match	TSVM prediction congruent?
1923	DGEDHGTV | | |:| DAESIGSV	BacB-M1-Lys bacitracin synthetase 2	No: val = leu = ile = abu = iva-like specificity
1924 mod1	DLTKIGHV ||||:||: DLTKVGHI	SrfAB-M2-Asp surfactin synthetase B	Yes: asp = asn = glu = gln = aad-like specificity
1924 mod2	DFWNIGMV |||||||| DFWNIGMV	PvdD-M2-Thr pyoverdine synthetase	Yes: thr = dht-like specificity
1925 mod1	DFWNIGMV |||||||| DFWNIGMV	PvdD-M2-Thr pyoverdine synthetase	Yes: thr = dht-like specificity
1925mod2	DVWHVSLI |||||||| DVWHVSLI	PvdJ-M1-Ser pyoverdine synthetase	Yes: ser-like specificity
1926 mod1	DLTKIGHV ||||:||: DLTKVGHI	SrfAB-M2-Asp surfactin synthetase B	Yes: asp = asn = glu = gln = aad-like specificity
1926 mod2	DVWHVSLI |||||||| DVWHVSLI	PvdJ-M1-Ser pyoverdine synthetase	Yes: ser-like specificity

### Mass spectrometry of pyoverdine purified from *P. syringae *1448a

**Figure 2:**
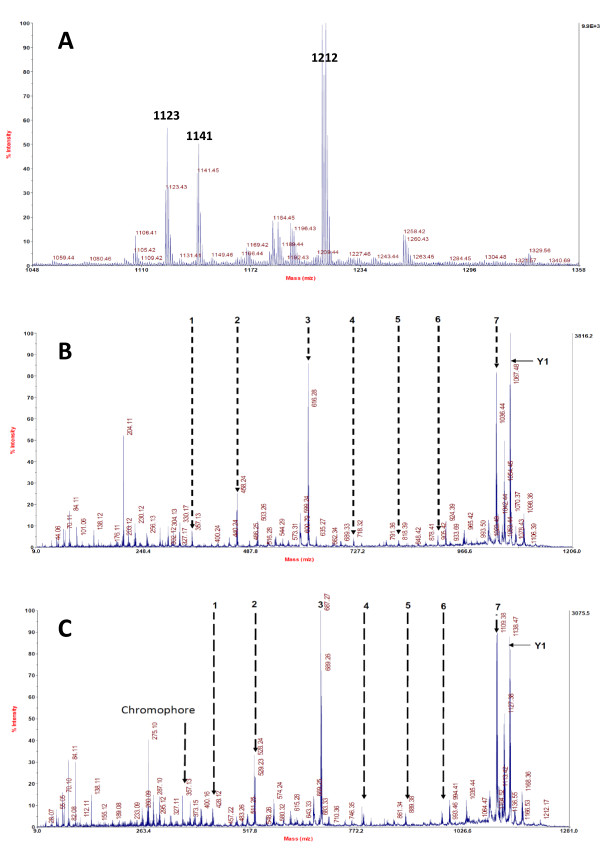
**Mass spectral analysis of pyoverdine purified from *P. syringae* 1448a**. A. MALDI-TOF analysis showing three major [M+H]+ species. Ions corresponding to cyclic (m/z = 1123) and linear (m/z = 1141) pyoverdine are present along with a third variant species (m/z = 1212). **B. **MS/MS analysis of m/z = 1141 precursor; masses and putative identity of indicated peaks are presented in Table 3. **C. **MS/MS analysis of m/z = 1212 precursor showing a set of fragment ions 71 Da heavier than those indicated in part B (masses presented in Table 4).

To test the *in silico *predictions above we purified the pyoverdine species secreted by *P. syringae *1448a using amberlite bead affinity chromatography as previously described [16]. Fractions were collected and analysed for siderophore activity by addition of chromeazurol S (CAS; a dye that is blue-green when complexed with iron and yellow when iron is removed from it) and the fraction with the highest activity was subjected to MALDI-TOF analysis to identify the mass of the primary constituents (Figure 2A). This revealed the presence of three major positive ion peaks. One of these peaks (m/z 1141) is consistent with the linear (hydrolysed) pyoverdine structure portrayed in Figure 1B, while another (m/z 1123) corresponds to the cyclized form observed in other *P. syringae *pathovars, in which an ester bond between the C-terminal carboxyl and the side chain of the second internal threonine residue results in a lactone structure [35]. The third peak (m/z 1212), 71 mass units greater than linear pyoverdine, could not be explained by either the *in silico *characterization above or by comparison with the structures previously elucidated for other *P. syringae *pathovars. We hypothesized that this peak resulted from either a pyoverdine molecule bearing an alternative acyl substituent attached to the chromophore (71 Da larger than the succinate-derived moiety portrayed in Figure 1B) or a contaminant that had co-purified with pyoverdine.

To test this hypothesis, and to investigate the identity and order of the amino acids present in the pyoverdine side chain, the peaks at m/z 1141 and 1212 were subjected to MS/MS analysis. Fragmentation of the peak at m/z 1141 resulted in the formation of a set of B ions (Figure 2B, Table 3) that corresponded exactly to the order and identity of amino acids predicted in Figure 1B. In contrast, fragmentation of the peak at m/z 1212 resulted in a series of peaks with identical spacing and intensity to those in Figure 2B, but 71 Da larger (Figure 2C, Table 4). This immediately discounted the possibility that the MALDI-TOF peak at m/z 1212 arose from sample contamination. Moreover, in both Figure 2B and 2C there are peaks at m/z 357 (Tables 3 and 4), corresponding to the predicted mass of the pyoverdine chromophore with an attached acyl group derived from succinate. In both spectra there are also intense peaks that correspond a Y-ion (marked Y1, Figure 2B, C) formed as a result of loss of the acyl group from the chromophore; and these peaks also differ by 71 Da. Together, these results suggest that the variant pyoverdine species differs from that portrayed in Figure 1B not in the structure of its chromophore, but rather in the constitution of its polypeptide side chain. Finally, we note that there is a fourth, smaller peak at m/z 1194 in the MALDI-TOF spectrum (Figure 2A), which may correspond to a cyclized form of this larger pyoverdine species.

**Table 3:** Negative ions arising from MS/MS analysis of the m/z = 1141 pyoverdine species

Peak number	Mass	Composition of ion
1	357.13	B ion: CHR
2	458.24	B ion: CHR_K
3	616.28	B ion: CHR_K_OH-D
4	718.32	B ion: CHR_K_OH-D_T
5	818.39	B ion: CHR_K_OH-D_T_T
6	905.42	B ion: CHR_K_OH-D_T_T_S
7	1036.41	B ion: CHR_K_OH-D_T_T_S_OH-D
Y1	1067.48	Y ion resulting from loss of chromophore acyl group

Fragmentation of the m/z = 1141 pyoverdine species resulted in identification of the following negative ions as shown in Figure 2B. Peaks 1-7 match the expected pattern of B-ions previously reported for fragmentation of other P. syringae linear pyoverdine molecules. Y1 has the expected mass for the Y ion resulting from loss of the acyl group of the chromophore. CHR = chromophore, OH-D = hydroxyaspartate, all other amino acids indicated by standard one letter code.

**Table 4:** Negative ions arising from MS/MS analysis of the m/z = 1212 pyoverdine species

Peak number	Mass	Mass difference with equivalent peak in Table 3
CHR	357.13	0
1	428.12	70.99
2	529.23	70.99
3	687.27	70.99
4	789.30	70.98
5	889.38	70.99
6	976.43	71.01
7	1107.40	70.99
Y1	1138.47	70.99

Fragmentation of the m/z = 1212 pyoverdine species resulted in identification of the following negative ions as shown in Figure 2C. The numbering and spacing of ions is identical to those listed in Table 3, but with peak 1 now representing the chromophore bearing an unknown 71 Da substituent. Y1 has the expected mass for the Y ion resulting from loss of the acyl group of the chromophore (allowing for the unknown 71 Da substituent).

### Genetic and biochemical analysis of the pyoverdine NRPS genes

**Figure 3:**
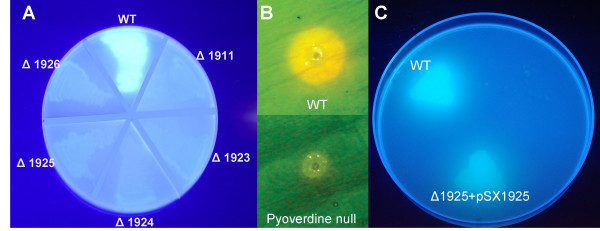
**Characterization of *P. syringae* 1448a pyoverdine NRPS knockouts**. **A. **Wild type (WT) and pyoverdine NRPS knockouts (Δ1911, Δ1923-1926) on iron-limiting KB agar viewed under UV light. Only the wild type is able to synthesize fluorescent pyoverdine. Pyoverdine gene knockout strains are named according to the gene deleted, based on the Pspph gene numbering scheme in the published genome database [27]. **B. **Wild type and pyoverdine null strain (Δ1925) inoculated into KB agar containing CAS dye and incubated for 24 h at 28°C. Only the wild type strain took up discernible levels of iron as evidenced by the orange halo surrounding this inoculum. All pyoverdine NRPS knockouts exhibited indistinguishable iron transport deficient phenotypes. **C. **Wild type, Δ1925 and Δ1925 complemented by pSX:1925 on iron-restricted KB agar containing 200 μg/ml EDDHA. Complementation by a functional gene copy in trans restored pyoverdine synthesis to near wild type levels in each of the NRPS knockout strains.

To confirm that each of the putative pyoverdine NRPS genes was indeed required for pyoverdine biosynthesis, these were individually deleted in-frame from the chromosome using a rapid overlap PCR-based method [37,38]. When grown on iron-limiting King's B (KB) media [39] each NRPS gene deletion strain lacked the UV fluorescence of wild type (WT) (Figure 3A). Likewise, each of the gene deletion strains was impaired in siderophore production, assessed following 24 h growth on CAS agar plates at 28°C (Figure 3B); and was unable to grow on KB agar plates containing 200 μg/ml EDDHA (ethylene-diamine-di-hydroxyphenylacetic acid, an iron chelating agent that establishes a strong selective pressure for effective siderophore-mediated iron transport; Figure 3C). These phenotypes confirmed that none of the gene deletion strains were able to produce pyoverdine. Successful restoration of pyoverdine synthesis by complementation *in trans *indicated that these phenotypes did not result from polar effects. Restoration of pyoverdine synthesis was demonstrated through the re-establishment of UV fluorescence and the ability to grow on KB agar plates containing 200 μg/ml EDDHA (Figure 3C), as well as a positive phenotype on solid and liquid media CAS assays (not shown).

To confirm the pyoverdine NRPS substrate specificity assigned by *in silico *analysis, and also to investigate the possibility that relaxed substrate specificity for one of the NRPS modules might explain the presence of a variant pyoverdine species, we sought to express and purify each side chain module as a heterologous His6-tagged protein from *Escherichia coli *for biochemical characterization. However we were unable to recover any proteins that were functional in substrate specificity assays, despite managing to obtain soluble protein for full modules as well as isolated A-domains by several different methods (including low temperature growth in the presence of 2.5 mM glycine betaine and 1 M D-sorbitol, a strategy that previously enabled us to isolate functional recombinant PvdD from *P. aeruginosa *PAO1 [19]; and over-expression and purification of recombinant proteins in the native *P. syringae *1448a host). In contrast, we were able to express and purify two functional single-module NRPS control proteins, EntF from *E. coli *and BpsA from *Streptomyces lavendulae *[40].

### Characterization of achromobactin as a secondary siderophore of *P. syringae *1448a

**Figure 4:**
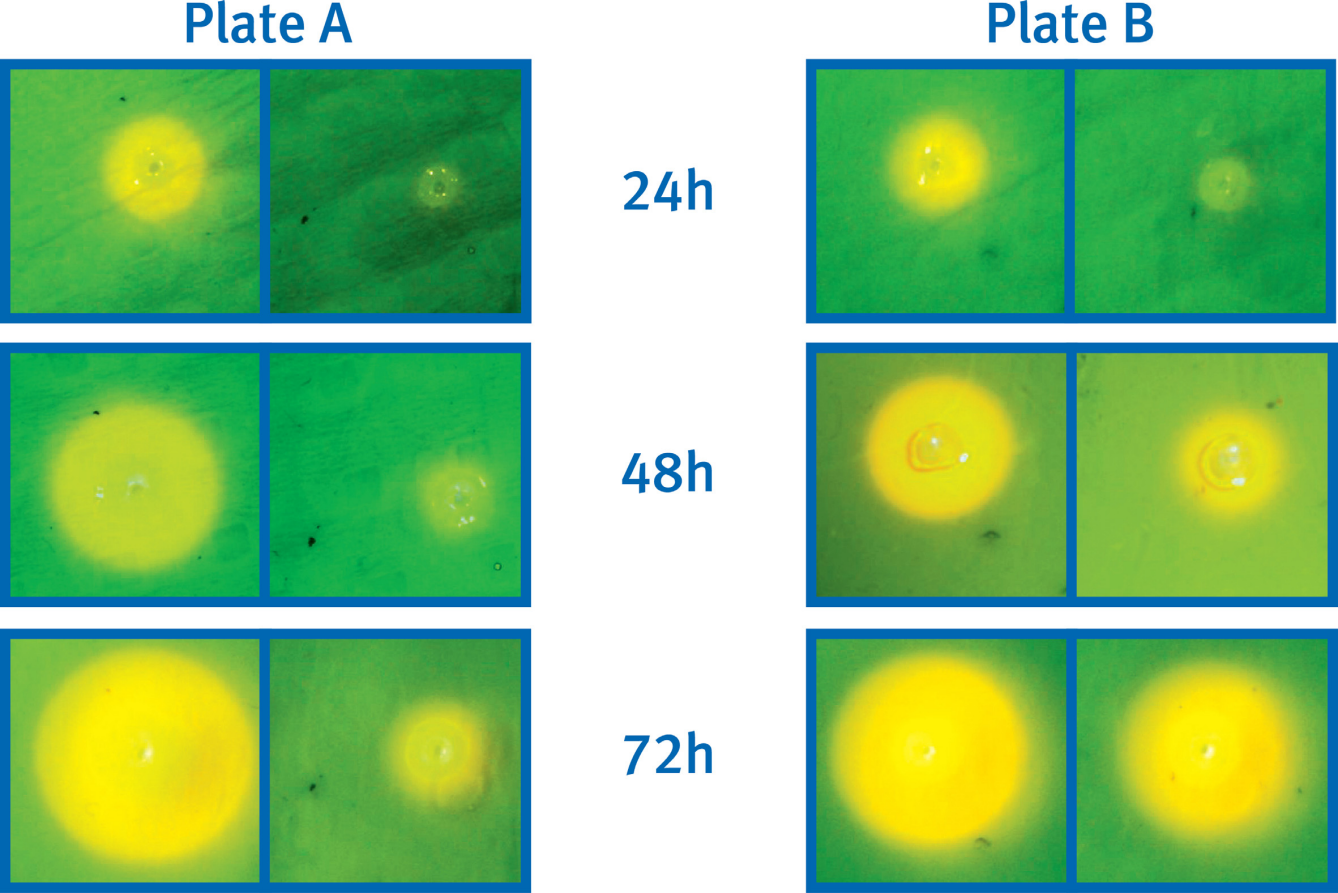
**Temperature-dependent production of a secondary siderophore by pyoverdine null *P. syringae *1448a**. Wild type and pyoverdine null *P. syringae* 1448a colonies were inoculated into identical Kings B plates containing CAS dye. Both plates were incubated at 28°C for 24 h, following which plate B was removed to 22°C for the remainder of the experiment while plate A was maintained at 28°C. For each plate, wild type is on the left, and the pyoverdine null strain is on the right.

Although the pyoverdine deficient (pvd^-^) strains were unable to discernibly alter the color of the CAS dye during 24 h growth on agar at 28°C (Figure 3B), i.e. no active iron sequestration was apparent within this timeframe, some color change was observed when these plates were subsequently left at room temperature or maintained at 28°C for an extended duration. These observations suggested that the pvd^- ^strains were secreting at least one alternative siderophore. Production of the secondary siderophore(s) appeared to be temperature dependent, with the pvd^- ^strains exhibiting greater iron uptake at 22°C than at 28°C (the latter being the optimal laboratory temperature for growth of *P. syringae *1448a [41]) (Figure 4A, B). However, none of the pvd^- ^strains were able to grow during 72 h incubation at either temperature on solid media containing 200 μg/ml EDDHA, indicating that the secondary siderophore(s) had much lower affinity than pyoverdine for iron.

To identify candidate genes governing synthesis of this secondary siderophore, some known siderophore synthetase sequences from other phytopathogenic bacteria were aligned by BLASTP against the *P. syringae *1448a genome [27,42]. This search revealed that *P. syringae *1448a contains gene clusters that are highly conserved (containing the same number and order of homologous genes) with the achromobactin biosynthetic locus of *P. syringae *pv. syringae B728a [20] and the yersiniabactin biosynthetic locus of *P. syringae *pv. tomato DC3000 [43].

To investigate the role of these gene clusters the *P. syringae *1448a *acsA *(achromobactin biosynthesis [20]) and *hmwp1 *(yersiniabactin biosynthesis [43]) homologs were deleted in-frame from both WT and pvd^- ^strains of *P. syringae *1448a. On solid media both the achromobactin (acr^-^) and yersiniabactin (ybt^-^) single mutants were indistinguishable in phenotype from wild type, growing effectively in the presence of 200 μg/ml EDDHA and rapidly taking up iron on CAS agar. In contrast, a pvd^-^/acr^- ^double mutant was unable to take up any discernible amounts of iron on CAS agar irrespective of the duration or temperature of incubation (after 72 h at either 22 or 28°C pvd^-^/acr^- ^colonies on CAS agar appeared identical to the 24 h pvd^- ^mutant pictured in Figure 3B). Using silica chromatography as previously described [20] we were able to isolate a siderophore from a culture of pvd^- ^*P. syringae *1448a grown to stationary phase in iron-limiting M9 minimal medium. When the fraction with the greatest siderophore activity (determined by addition of CAS dye) was analysed by MALDI-TOF, major peaks at m/z 590.2 and 572.2 were detected (not shown). The larger peak is consistent with the published mass for achromobactin of 590.15 Da [20]; while the smaller peak most likely represents the same species following loss of a water molecule - when the same fraction was evaporated to dryness then resuspended in solvent prior to analysis, the relative intensity of the peak at m/z 572.2 substantially increased.

Surprisingly, despite appearing to have the genetic potential to make yersiniabactin, *P. syringae *1448a does not appear to produce any high-affinity siderophores other than pyoverdine and achromobactin. We were unable to observe any secretion of yersiniabactin by the pvd^-^/acr^- ^double mutant and a pvd^-^/acr^-^/ybt^- ^triple mutant was indistinguishable from the pvd^-^/acr^- ^double mutant in all phenotypic assays conducted in this work. To test whether laboratory passage of our *P. syringae *1448a strain might have resulted in inactivation of the yersiniabactin genes by phase-shifting or another reversible mechanism, we repeatedly sub-cultured the pvd^-^/acr^- ^double mutant in iron-limiting KB broth on a daily basis for 7 days, each day plating out a dilution that gave ca. 10^3 ^colonies on CAS agar. Duplicate plates were incubated at either 22°C or 28°C for up to 72 h, but no siderophore-secreting colonies were recovered. We therefore concluded that *P. syringae *1448a produces only two high-affinity siderophores in response to iron deprivation, pyoverdine and achromobactin.

**Figure 5:**
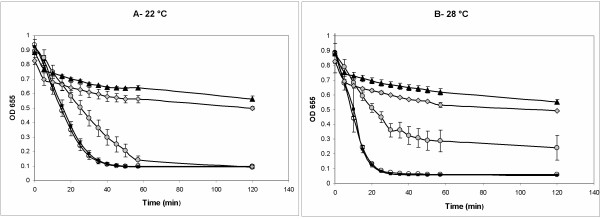
**Liquid CAS assay**. 96-well plate wells containing 200 μl unamended King's B liquid media were inoculated in triplicate from synchronized overnight cultures of the following strains: WT (black squares), acr- (white circles), pvd- (grey circles), and pvd-/acr- (grey diamonds). A triplicate media-only control (black triangles) was also included. Plates were incubated with shaking at either 22°C (**A**) or 28°C (**B**) for 48 h. Cells were then pelleted and 150 μl supernatant removed to fresh wells. CAS dye (30 μl) was added to each well and the rate at which iron was removed from the dye by secreted factors in the supernatant was followed at OD 655 (monitoring loss of blue coloration). Error bars are presented as ± 1 standard deviation.

When each of the WT, pvd^-^, acr^-^, and pvd^-^/acr^- ^strains were grown in liquid media and subjected to a modified CAS assay that we developed to measure iron acquisition by factors secreted into the culture supernatant, the results were consistent with the phenotypes observed for each strain on CAS agar (Figure 5). These results confirmed that *P. syringae *1448a is able to employ achromobactin as a temperature-regulated secondary siderophore that is secreted into the extracellular environment for active uptake of iron; but also suggested that the presence of pyoverdine is able to mask any phenotypic effects due to achromobactin alone.

### Assessment of relative fitness of mutant strains under iron starvation conditions

To more precisely quantify the contribution of each siderophore under varying degrees of iron starvation, a serial dilution experiment was performed, employing EDDHA concentrations diluted 1:2 from 800 μg/ml down to 0.2 μg/ml in KB media in a 96-well plate. The WT, pvd^-^, acr^-^, and pvd^-^/acr^- ^strains were replica-inoculated into each well and incubated with shaking at 22°C for 24 h, following which culture turbidity was measured. IC_50 _values (indicating the concentration of EDDHA that yielded only 50% turbidity relative to the unchallenged control) were calculated for each of the strains using Sigma Plot. The IC_50 _for the WT (260 ± 50 μg/ml) and acr^- ^(220 ± 70 μg/ml) strains were approximately equal, confirming that pyoverdine is able to compensate for achromobactin deficiency. In contrast the pvd^- ^strain was sensitive to almost 3 orders of magnitude less EDDHA, with an IC_50 _of only 0.57 ± 0.02 μg/ml, demonstrating that achromobactin cannot completely compensate for the absence of pyoverdine. However, the IC_50 _for the pvd^-^/acr^- ^double mutant strain (0.31 ± 0.01 μg/ml) was reproducibly lower yet, verifying that in the absence of pyoverdine achromobactin still makes a small contribution to fitness during iron starvation. At 28°C the IC_50 _for WT and acr^- ^strains were essentially unchanged, but the difference between the pvd^- ^mutant (0.38 ± 0.01) and pvd^-^/acr^- ^double mutant (0.26 ± 0.01) was less marked.

### Assessment of pathogenicity in *Phaseolus vulgaris*

**Figure 6:**
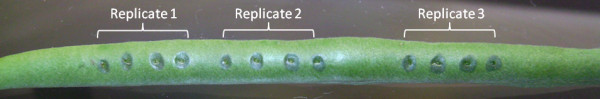
**Assessment of pathogenicity of mutant strains in *Phaseolus vulgaris***. Three replicates are indicated each containing, in order from left to right, WT, pvd-, acr/pvd- and acr-/pvd-/ybt- strains. Each strain was inoculated from a single colony, using a hypodermic needle. The pod was then incubated in a humid chamber at room temperature for 48 hours. All strains display characteristic water-soaked lesions indicating successful establishment of pathogenicity in *Phaseolus vulgaris.*

In order to assess the pathogenicity in the natural host of *P. syringae *1448a each of the mutant strains (including the pvd^-^/acr^-^/ybt^- ^triple mutant) was subjected to the standard 'bean prick' pathogenicity test using bean pods [44]. All mutant strains were still able to cause characteristic water soaked lesions after inoculation and incubation in bean pods (Figure 6), irrespective of temperature and whether or not the beans were picked or still attached to the parental plant. This indicates that neither pyoverdine nor achromobactin is essential in enabling *P. syringae *1448a to cause halo blight in the bean plant *Phaseolus vulgaris*.

## Discussion

Unlike *P. aeruginosa, P. syringae *does not appear to exhibit a high degree of variability in pyoverdine structure from strain to strain, with all fluorescent *P. syringae *pathovars tested thus far having been found to produce an identical pyoverdine molecule [35,36]. Our bioinformatic studies suggested that *P. syringae *1448a would not be any different in this regard; and MALDI-TOF and MS/MS analyses demonstrated that the same pyoverdine is indeed made by this strain. However, these analyses also indicated that *P. syringae *1448a is able to make an additional pyoverdine variant that was fundamentally similar in most aspects, but with an overall mass 71 Da greater.

The most plausible interpretation of the fragmentation pattern in Figure 2C is that an extra monomer is incorporated into the pyoverdine side chain. If so, the B-ion pattern suggests that this monomer appears as the first residue of the side chain, falling between the chromophore and L-lysine, and increasing the mass by 71 Da. The only amino acid which could give this mass increase is alanine (free molecular mass of 89 Da; 71 Da post-condensation). However, *in silico *analysis of all NRPS modules present in the genome of *P. syringae *1448a failed to reveal any A-domains predicted to specify alanine. One possibility may be that the variant pyoverdine species was generated as an artefact of the purification process through some unexplained mechanism; however, as the additional monomer clearly seems to fall between the chromophore and lysine residue rather than being added in a peripheral fashion, this explanation seems unlikely. An alternative explanation is that the product of *P. syringae *1448a gene *Pspph*1923 (the single-module NRPS predicted to incorporate L-lysine; Table 2) may possess a dual activity that enables occasional incorporation of an additional alanine residue. Unfortunately we were unable to biochemically characterize the substrate specificity of this or any other of the pyoverdine NRPS modules in *in vitro *assays - despite obtaining soluble protein by several different strategies, none of our purified proteins appeared to retain activity. This phenomenon is not uncommon for NRPS enzymes. We note however that in ongoing work we have verified the second module of *Pspph*1925 is indeed a serine-activating NRPS, as predicted by our *in silico *analysis (Table 2); when appropriate regions of this gene are swapped with the equivalent regions in module 2 of *P. aeruginosa *PAO1 *pvdD *the substrate specificity of the recombinant gene product is converted from L-threonine [19] to L-serine, and a correspondingly modified pyoverdine product is produced (MJ Calcott, JG Owen, LW Martin, IL Lamont, DF Ackerley, unpublished data). It may be that we can employ a similar 'recombinant genetic characterization' strategy to interrogate the substrate specificity of *Pspph*1923. However, for now the precise nature of the variant *P. syringae *1448a pyoverdine species (peak m/z 1212, Figure 2A) remains unknown. Although an equivalent species was not previously detected in studies of other *P. syringae *pathovars [35,36], it is possible that these other pathovars also produce this form. As MALDI-TOF is not a quantitative technique the m/z 1212 peak may actually be a very minor species that happens to ionize particularly well; and as the previous studies utilized an HPLC preparative step to yield a single pure peak, this could conceivably have resulted in other minor peaks being missed. There is evidence from a previous isoelectric focusing analysis that different *P. syringae *pathovars produce minor variant isoforms of pyoverdine in addition to the major pyoverdine that is synthesized by all known fluorescent *P. syringae *isolates [45]. It is possible that the minor isoforms include variants that possess alternative side chain constituents as well as variants that have different acyl groups attached to the chromophore.

As per previous pyoverdine NRPS gene knockouts in fluorescent pseudomonads [16,46], in-frame deletion of any of the chromophore or side chain NRPS genes in *P. syringae *1448a resulted in complete abolition of pyoverdine synthesis. Analysis of these mutants under iron-limiting conditions revealed the presence of a secondary siderophore, which was shown by genetic and biochemical analysis to be achromobactin. Although *P. syringae *1448a also appears to have the genetic potential to produce a third siderophore, yersiniabactin, our pvd^-^/acr^- ^double mutant did not appear to be able to make this or any other siderophores, at least in response to iron limitation. Our study does not rule out that yersiniabactin synthesis might be induced in *P. syringae *1448a *in planta*, but this would contrast with yersiniabactin synthesis in *P. syringae *pv. tomato DC3000, which occurs both *in planta *[46] and under iron-limiting conditions *in vitro *[43].

We observed that synthesis of achromobactin by our pvd^- ^mutant was temperature sensitive. Temperature regulation of siderophore production has been observed for other bacterial species [47-49] and has been known to govern expression of other *P. syringae *genes, especially those implicated in causing disease [50]. Achromobactin is known to contribute to virulence in *D. dadantii *[25], and these observations prompted us to test whether it is a virulence factor in *P. syringae *1448a also. The contribution of both achromobactin and pyoverdine to virulence of *P. syringae *1448a during infection of *Phaseolus vulgaris *was assessed by inoculation of mutant strains and wild type controls into the bean pods. All single and double mutants were still able to cause lesions in this standardized pathogenicity test, indicating that neither siderophore is required for *P. syringae *1448a to cause halo blight in *Phaseolus vulgaris*. These results were initially surprising to us, given that iron is essential for core metabolic processes, is believed to be severely restricted in the plant extracellular environment [51], and that siderophores are generally regarded as important for microbial pathogenesis of both plant and animal hosts [6,51]. However, although the assumption is frequently made that pyoverdines are able to act as virulence factors in both animal and plant hosts, there is little experimental evidence for the latter. Indeed, pyoverdine from *P. syringae pv*. syringae has likewise been shown not to have a determinative role in pathogenesis of sweet cherry fruit [52] and more recently, pyoverdine in *P. syringae *pv. tomato DC3000 has also been shown to be dispensable for pathogenesis [46]. It may be that phytotoxins render siderophores obsolete during the disease process by releasing iron from damaged plant cells into the extra-cellular environment. It should also be noted that the standard bean inoculation assay for *P. syringae *1448a virulence monitors only the ability to cause lesions, which is dependent primarily on toxin release and may not accurately report on the full progression of disease. Irrespective, it must be considered that any plant protection strategy which aims to target pyoverdine and/or achromobactin in *P. syringae pv. phaseolicola *will not prevent the appearance of economically-damaging halo blight lesions in bean crops.

Despite the lack of evidence for an active role in lesion formation, our phenotypic analyses of iron uptake and growth under iron limiting conditions confirmed that siderophores are indeed important for fitness of *P. syringae *1448a during iron starvation. Although *P. syringae *has traditionally been defined as a phytopathogen, it is unclear how important pathogenicity really is to the survival of this bacterium in the wild [53]; and it may be that the *P. syringae *1448a siderophores are more important for epiphytic survival on leaf surfaces, in soil or water than during infection. However, given the clear superiority of pyoverdine as a siderophore, it is unclear why *P. syringae *1448a makes achromobactin also. All of the fluorescent *Pseudomonas *species known apart from one exception (*P. putida *KT2440 [54]) synthesize at least one secondary siderophore and there is presumably some fitness benefit to be derived from this investment. There is evidence that secondary siderophores can have affinity for metals other than iron (reviewed by Cornelis [55]). The presence of orthologs of known nickel-transport genes immediately adjacent to the *P. syringae *1448a achromobactin cluster in the *P. syringae *1448a genome sequence [27] may be indicative of a similar role in this bacterium (although we were unable to discern any phenotypic effect of nickel addition or exclusion on achromobactin synthesis in the pvd^- ^mutant; not shown). It has also recently been shown that both primary and secondary siderophores (including the pyoverdine and pyochelin produced by *P. aeruginosa *[56]) can actually play defensive roles in sequestering toxic metals like aluminium, cobalt, copper and lead, which appears to protect bacteria against uptake of these metals by passive diffusion [57]. Independent of a direct role in metal transport or sequestration, it has been suggested that secondary siderophores can also be involved in various signaling pathways [55], or can have antimicrobial activities that are distinct from their iron scavenging properties [58].

Alternatively, Dominique Expert and co-workers have demonstrated that achromobactin in the phytopathogen *D. dadantii *is synthesized temporally before the primary NRPS-derived siderophore chrysobactin [25]; and have proposed that achromobactin in this bacterium may function as a provisional measure, enabling cells to respond more rapidly to fluctuations in iron availability while the slower chrysobactin system is established [25,51]. We suggest that a likely explanation for this scenario lies with the high energy investment required for activating NRPS mechanisms of siderophore synthesis. NRPS enzymes are amongst the largest known, with single proteins routinely exceeding 200 kDa [59]. The energy requirements for a cell to synthesize such large proteins are substantial, and when already stressed this may represent a formidable barrier. However, once the NRPS enzymatic template is in place then it is an extremely efficient method for synthesizing short peptides, consuming significantly less ATP per peptide bond formed than ribosomal mechanisms [60]. It might therefore be useful to have a backup siderophore in place that can be expressed immediately in response to iron starvation and provide the cell with small amounts of iron while the NRPS template for the more efficient primary siderophore is established. As the phenotypes of our mutant strains indicate that achromobactin is only important when pyoverdine is not available, it is possible that achromobactin likewise serves as a 'first response' siderophore to cope with a sudden onset of iron starvation in *P. syringae *1448a. Our investigation into the timing and regulation of pyoverdine and achromobactin synthesis in *P. syringae *1448a is ongoing.

## Conclusions

*P. syringae *1448a appears to have the genetic capacity to produce three different siderophores however only two of these, pyoverdine and achromobactin, were detectable as active siderophores under the various conditions examined. An essential role for five NRPS genes in pyoverdine synthesis was confirmed by gene deletion and complementation studies, and the *in silico *assignation of substrate specificity for each NRPS module was found to be congruent with a structure for *P. syringae *1448a pyoverdine inferred from MS/MS data. Surprisingly, this data also indicated that *P. syringae *1448a produces a second, heavier, isoform of pyoverdine, which may contain an extra alanine residue located between the chromophore and the lysine residue of the peptide side chain. Although pyoverdine was shown to be a substantially more effective siderophore than achromobactin, neither siderophore was found to play a definitive role in the ability of *P. syringae *1448a to cause halo blight, indicating that these siderophores are not promising targets for development of novel antibiotics to protect bean crops.

## Methods

### Bioinformatics and computer programs

Adenylation domain specificities for putative pyoverdine NRPS modules were predicted using the NRPS/PKS predictor currently online at http://nrps.igs.umaryland.edu/nrps/, based on the 8 amino acid model of A domain prediction [32]. Specificities were also predicted using the TSVM method [33] with congruent results. For analysis of the pyoverdine cluster of *P. syringae *1448a, inferred amino acid sequences of known pyoverdine genes from *P. aeruginosa *PAO1 (as described in [6,8]) were aligned against the *P. syringae *1448a genome using the default BLASTP settings of the *Pseudomonas *genome database http://www.pseudomonas.com[27]. Genes were taken to be orthologs if they were annotated as being in the same COG group; up to 5 matches were recorded where orthologous genes were not clearly present in the known pyoverdine locus and/or had a shared amino acid identity under 40%. Annotated hits were then mapped onto the corresponding section of the *P. syringae *1448a chromosome, derived from the *Pseudomonas *genome data base. This map was compared for accuracy against the map presented by Ravel and Cornelis [8], updated to include more-recently discovered *pvd *genes, and a simplified version was used to generate Figure 1. The pyoverdine structure for *P. syringae *1448a was adapted from Bultreys et al [35] and recreated and re-colored using the GIMP open office image manipulation software. Achromobactin and putative yersiniabactin genes were identified by BLASTP searching against the *P. syringae *1448a genome using the corresponding protein sequences from *D. dadantii *[25] and *P. syringae *pv. tomato DC3000 [43], respectively. The putative function of the genes immediately surrounding the achromobactin cluster was derived from the annotations in the *Pseudomonas *genome database.

### Bacterial strains, growth and maintenance

The following bacterial strains were utilized in this study: rifampicin-resistant *P. syringae *1448a, kindly provided by Professor John Mansfield [61]; and *E. coli *DH5α λpir (Invitrogen). *P. syringae *1448a was routinely maintained at 28°C using LB or KB media. *E. coli *strains were maintained at 37°C using LB media. Aeration of liquid cultures was provided by shaking at 200 rpm. When necessary for plasmid or chromosomal antibiotic marker selection antibiotics were used at the following concentrations: rifampicin 50 μg/ml, chloramphenicol 35 μg/ml, gentamycin 20 μg/ml.

### Purification and analysis of pyoverdine

Pyoverdine purification was achieved using the method of Meyer et al [62]. Briefly, 200 ml of standard M9 minimal medium, with succinic acid as the carbon source, was inoculated with 10 ml *acr*^- ^*P. syringae *1448a from a stationary phase culture grown in the same medium. The resulting culture was grown for 72 h (22°C, 200 rpm) following which cells were removed by centrifugation (5000 g, 30 min). The supernatant was then sterilised by passing through a 0.22 μm filter and the pH of the resulting 200 ml culture supernatant adjusted to 6.0 with cHCl. Approximately 40 cc wet Amberlite XAD-4 resin (Supelco, PA), which had been previously activated according to the manufacturer's directions, was added to the acidified culture supernatant. The mixture was then shaken for 90 min at 200 rpm, after which the beads were discernibly green, indicating pyoverdine adsorption. The supernatant was then discarded and the beads washed five times with 200 ml ddH_2_O, shaking at 200 rpm for 15 min. After this the beads were washed with 500 ml ddH_2_O (5 min, 200 rpm), then 500 ml of 15% v/v methanol (5 min, 200 rpm). Pyoverdine was then removed from the beads by shaking with 100 ml of 50% v/v methanol (200 rpm, 2 h) and the resulting solution freeze-dried. Purified pyoverdine was resuspended in 1 ml ddH_2_O and, following confirmation of siderophore activity by CAS assay, sent to the Centre for Protein Research at the University of Otago for MALDI-TOF and MS/MS analysis.

### Purification and analysis of achromobactin

The protocol for achromobactin purification was adapted from Berti and Thomas [20]. Briefly, 200 ml of standard M9 minimal medium, with succinic acid as the carbon source, was inoculated with 10 ml *pvd*^- ^*P. syringae *1448a from a stationary phase culture grown in the same medium. The resulting culture was grown for 72 h (22°C, 200 rpm) following which cells were removed by centrifugation (5000 g, 30 min). The supernatant was then sterilised by passing through a 0.22 μm filter and then the volume reduced to 20 ml by rotary evaporation (temperature not exceeding 45°C). Methanol (180 ml) was then added, whereupon salt from the culture medium precipitated out of solution. Precipitate was removed by centrifugation (12,000 rcf, 20 min) followed by filtration using a 0.45 μm filter. The solution was then mixed 1:1 with ethyl acetate and 100 ml of the resulting solution applied to a glass chromatography column containing 40 cc silica beads pre-equilibrated with solvent A (9:1:10 v/v methanol:H_2_O:ethyl acetate). 100 ml Solvent A was then applied to the column, followed by 100 ml solvent B (9:1 v/v methanol:H_2_O). The elutate from the solvent B step was captured in 10 ml fractions. Siderophore activity of the fractions was then assessed by adding 30 μL CAS reagent to a 150 μL aliquot of each fraction and incubating for 10 min at room temperature. The fraction which resulted in the greatest discolouration of the CAS dye was then reduced in volume to 2 ml by rotary evaporation (temperature not exceeding 40°C) and 1 ml of the solution removed. The remaining 1 ml was evaporated to dryness and resuspended in 1 ml ddH_2_O. Both of these 1 ml samples were then sent to the Centre for Protein Research at the University of Otago for MALDI-TOF analysis.

### Construction of gene knockout and over-expression plasmids

Gene sequences were retrieved from the *Pseudomonas *genome database [27]. Primers were designed using Vector NTI (Invitrogen) to amplify 400 bp regions from the 5' and 3' regions of the NRPS genes (including the putative yersiniabactin cluster gene *hmwp1*) such that when they were fused no frame shift would result (all primers used in this study are listed in Additional file 1, Table S1). For deletion of *acsA*, which is much smaller, 400 bp regions immediately upstream and downstream of the gene, including the first and last 3 codons of the gene on either side, were amplified. The upstream primer of the 3' fragments contained a region complementary to the downstream primer of the 5' fragment for use in splice overlap extension (SOE) PCR [38]. The outer-most primers contained restriction enzyme sites to enable directional cloning of the spliced fragments into the suicide vector pDM4 [63], following which gene knockout was performed as described below.

For gene complementation studies we generated an IPTG-inducible broad-host range vector, pSX, by cloning the *lacIQ *gene, *tac *promoter and multiple cloning region of pMMB67EH [64] together with an artificially-introduced ribosome binding site into pUCP22 [65]. The full sequence of this plasmid is available on GenBank (accession number JN703735). *Pspph*1925 was PCR-amplified using the primers 1925compFw and 1925compRv (Supplementary Table 1) and directionally cloned into pSX via the introduced NdeI and HindIII restriction sites. The accuracy of this and all other plasmid gene inserts was validated by sequencing (Macrogen, Korea).

### Targeted deletion of *P. syringae *1448a genes

Mutagenic plasmids were delivered to *P. syringae *1448a using an electroporation protocol for *Pseudomonas *mutagenesis adapted from [38]. Overnight cultures were grown to stationary phase in LB media, then 6 ml of culture were aliquoted into 1.5 ml microfuge tubes for each electroporation. Cells were twice pelleted by centrifugation followed by resuspension in sterile 300 mM sucrose to wash. After the final wash all cells were pelleted, resuspended and pooled in 100 μl of 300 mM sucrose and transferred to a 2 mm gap electroporation cuvette together with 10 μl of mutagenic plasmid sample in ddH_2_O. Following electroporation and recovery as described [66], 100 μl samples were plated on LB containing chloramphenicol and rifampicin (*P. syringae *1448a is rifampicin resistant; this antibiotic was added to avoid growth of contaminants, not for selection of pDM4 chromosomal integrants). Plates were then incubated for 48-72 h at 28°C. Subsequent selection of primary integrants and *sacB *counter-selection were performed as previously described [38], with the resulting colonies screened for desired mutation events by colony PCR. For pyoverdine NRPS knockouts, mutant genotypes were also confirmed by Southern blotting using an Amersham alkphos^® ^kit with CDP Star^® ^detection reagent according to the manufacturer's instructions.

### CAS agar assays for iron uptake

100 ml Chromeazurol S (CAS) dye for the detection of siderophores [67] was made by dissolving 60.5 mg CAS powder (Sigma) in 50 ml distilled water. To this 10 ml of a 1 mM solution of FeCl_3 _was added. The entire solution was then poured slowly with stirring into 40 ml distilled water containing 72.9 mg dissolved HDTMA (Sigma) and autoclaved to sterilize. To make agar plates, freshly autoclaved KB agar was cooled to 60°C before adding 1 part CAS dye to 9 parts media. Plates were immediately poured, and at this point exhibited a dark green color. Strains were inoculated into dried CAS plates by picking a large colony with a sterile 100 μl pipette tip and piercing the tip approximately 5 mm into the surface of the agar plates. Plates were then incubated upside down at 28°C for 24 h. After 24 h incubation the 22°C condition was removed from the incubator and maintained at 22°C. Plates were photographed with minimal exposure to temperature change at 24, 48 and 72 h. The entire assay was repeated three times; results presented in figures are from a single assay and are representative of all repeats.

### CAS media assays for iron uptake

Strains were inoculated in triplicate into 200 μl KB media in a 96-well plate to an initial OD600 of 0.1, with outer wells filled with sterile H_2_O to minimize evaporation. Replicate plates were then covered but not sealed and incubated for 24 h at 28°C or 22°C with shaking. The next day cells were pelleted by centrifugation (4000 g, 15 min) and 150 μl of supernatant was transferred to fresh wells in a flat bottomed 96-well plate. To each well 30 μl of CAS dye (prepared as described above) was added using a multi channel pipette. Plates were immediately placed into the plate reader and OD 655 values recorded every 5 min for 50 min, then again at 65 min and 125 min.

### EDDHA Inhibitory Concentration (IC_50_) assays

A 2-fold serial dilution series of KB media containing from 200-0.195 μg/ml of the iron chelator EDDHA (ethylene-diamine-di(o-hydroxyphenylacetic acid); a generous gift from Dr Iain Lamont) was established in 96 well plates. Strains were inoculated in quadruplicate to an initial OD 600 of 0.1 from cultures synchronized by sub-inoculation over two nights, giving a final volume of 125 μl per well. Unsealed plates were then incubated for 24 h at 28°C or 22°C with shaking. Wells were diluted 1:1 with KB in order to be within the linear range of the plate reader, and OD 600 values were measured. For each temperature the assay was repeated twice with consistent results. Errors are presented as ± 1 standard deviation.

### *P. syringae *1448a pathogenicity tests in *Phaseolus vulgaris*

Single colonies from fresh 48 h KB agar plates were picked using a sterile hypodermic needle. Strains were then inoculated into snap bean pods (*Phaseolus vulgaris*) by piercing the surface of the bean approximately 5 mm. Each strain was inoculated in triplicate together with a WT positive control. Bean pods were then placed in a sealed humid containers or alternatively, for on plant assessment, pods were left attached to parental plants growing indoors at 20-25°C. Results were recorded every 24 h. Development of water soaked lesions similar to those of WT strain was taken as a positive result. The assay was repeated in triplicate.

## Authors' contributions

JGO co-designed the project, conducted the majority of the hands-on experimental work, and helped to draft the manuscript. DFA was the primary investigator and co-designed the project, assisted with experimental work, offered technical advice, obtained all funding, and drafted the manuscript. Both authors read and approved the final manuscript.

## Supplementary Material

Additional file 1 **Table S1 - supplementary table of PCR primers employed in this study**. A complete listing of all PCR primers employed in this work. Click here for file
