# Supplementary material for: Characterization of pyoverdine and achromobactin in Pseudomonas syringae pv. phaseolicola 1448a
Source: BMC Microbiol. 2011 Oct 3;11:218. doi: 10.1186/1471-2180-11-218 (PMC3207962; doi:10.1186/1471-2180-11-218)
Supplement: Additional file 1 — Table S1 - supplementary table of PCR primers employed in this study. A complete listing of all PCR primers employed in this work. [file 1471-2180-11-218-S1.DOCX]

# Additional files

### Additional file 1 – supplementary table of PCR primers employed in this study

| **Primer Name** | **Sequence** |
| --- | --- |
| 1911_Lup | GGGGCTCGAGGCCTTGCACACGCCGGATC |
| 1911_Ldwn | AGACGCACTGTCCAGCGTATCG |
| 1911_Rup_Hom | **cgtcgatacgctggacagtgcgtct**AGCGTGCCGGTGTTCGTG |
| 1911_Rdwn | CCCCTCTAGACTTGCGGTCAAGCTTGCC |
| 1923_Lup | GGGGGGATTCGATGTACGTGCGTAACTACGGC |
| 1923_Ldwn | GTTCATGGGTCGATCCTTGC |
| 1923_Rup_Hom | **ATTCAGCAAGGATCGACCCATGAAC**GCATGACCGGGACCACCG |
| 1923_Rdwn | GGGGGGATCCTTCGGTTTCAGCACGGCCG |
| 1924_Lup | GGGGCTCGAGATGACCGGGACCACCGCT |
| 1924_Ldwn | AGCCAGACCGGACAAATCAATG |
| 1924_Rup_Hom | **cagcattgatttgtccggtctggct**CTGAAGACCGAGCTCCGTGC |
| 1924_Rdwn | CCCCTCTAGAGTCCAGCGCATGGTCACTTTC |
| 1925_Lup | GGGGGGAGCTCACAGTTGCTTGACTCCGTTA |
| 1925_Ldwn | GCACTTCCTGATCGACATAA |
| 1925_Rup_Hom | **GAGCGTTATGTCGATCAGGAAGTGC**AAACACTGCCCGATTACAT |
| 1925_Rdwn | GGGGGAAGCTTTTTTTCAATATCGGACAAGC |
| 1926_Lup | GGGGCTCGAGATGGACAAGTCTGCAGCAGAGC |
| 1926_Ldwn | TTCGATGTCGGCTGGCGC |
| 1926_Rup_Hom | **ctcggttgcgccagccgacatcgaa**GTGGGGCTTATCGAAACCGC |
| 1926_Rdwn | CCCCTCTAGATCACACAAGCCGCTCGGC |
| AcsA_Lup | ggggCTCGAGATGAACTTCACTTCACTCGCCG |
| AcsA_Ldwn | CTCCAGAAACAGCGCTATGCC |
| AcsA_Rup_Hom | **aaagggcatagcgctgtttctggag**TGGCTTGAGCGTAACGGCA |
| AcsA_Rdwn | ggggTCTAGATCAGGTTGCACGGCGCAA |
| hmwp1_Lup | ccccctcgagATGTACACATCGACCAGAAAAGC |
| hmwp1_Ldwn | CTGATACAGATGACAGCC |
| hmwp1_Rup_Hom | **tggagtcggctgtcatctgtatcag**CTGGCTGAGCAACCCCAG |
| hmwp1_Rdwn | cccctctagaTCATGAACCACCTCCGCT |
| 1925compFw | CCCCCATATGCAACAGTTGCTTGACTCCG |
| 1925compRv | CCCCAAGCTTTCAGATACCCGCCATTTCATTC |

**Lup**-Forward primer for amplification of 5’ gene fragment for in-frame gene knockout.

**Ldwn**-Reverse primer for amplification of 5’ gene fragment for in-frame gene knockout.

**Rup**-Forward primer for amplification of 3’ fragment for in-frame gene knockout; contains 25 bp homology region to corresponding 5’ fragment for SOE PCR (bold).

**Rdwn**-Reverse primer for amplification of 3’ gene fragment.

Restriction sequences are underlined and homology regions for SOE PCR are highlighted in bold.
